# Plasma and fecal zonulin are not altered by a high green leafy vegetable dietary intervention: secondary analysis of a randomized control crossover trial

**DOI:** 10.1186/s12876-022-02248-3

**Published:** 2022-04-12

**Authors:** Aaron J. Riviere, Kristen S. Smith, Megan N. Schaberg, Michael W. Greene, Andrew D. Frugé

**Affiliations:** 1 Department of Nutrition, Dietetics, and Hospitality Management, Auburn University, Auburn, AL 36849 USA; 2 Department of Health and Kinesiology, Texas A&M University, College Station, TX 77843 USA

**Keywords:** Diet, Zonulin, Intestinal permeability, Microbiome

## Abstract

**Background:**

Zonulin is observed in animal models to regulate intestinal permeability and influenced by dietary intake, gut microbiota, and inflammation. We conducted a secondary analysis of a randomized controlled crossover trial (NCT03582306) in individuals with a BMI greater than 30 kg/m^2^ and high habitual red meat intake and low habitual green leafy vegetable (GLV) intake.

**Methods:**

Participants were provided with frozen GLV during the first or last four weeks (immediate or delayed intervention) of the twelve-week trial. Biological and anthropometric measures were taken at the beginning and at each four-week interval. A subset of 20 participants was selected for this secondary analysis of the intestinal permeability and inflammation-related biomarkers: serum and fecal zonulin; serum lipopolysaccharide binding protein (LBP), Alpha-1-acid glycoprotein 1 (ORM-1), tumor necrosis factor α (TNFα), interleukin-6 (IL-6), and C-reactive protein; 8-hydroxy-2'-deoxyguanosine (8OHdG) and plasma Vitamin K1 as a marker of protocol adherence. Nutrient and food group intake from two-24-h dietary recalls collected at each time point were assessed. Fecal microbiota was measured by 16 s rRNA PCR sequencing. Changes in biological markers, dietary factors, and microbial taxa were assessed with Wilcoxon Sign Ranks Tests. Exploratory analyses of the relationship between changes in outcome variables were conducted with Spearman correlations.

**Results:**

No changes in serum and fecal zonulin and serum LBP were observed. Plasma Vitamin K (*p* = 0.005) increased, while plasma 8OHdG (*p* = 0.023) decreased during the intervention compared to the control. The only dietary factors that changed significantly were increases during intervention in Vitamin K and Dark GLV (*p* < 0.001 for both) compared to control. Fecal microbiota did not change significantly across all times points; however, change in serum zonulin was associated with change in Proteobacteria (ρ = − 0.867, *p* = 0.001) in females and *Bifidobacterium* (ρ = − 0.838, *p* = 0.009) and *Bacteroidaceae* (ρ = 0.871, *p* = 0.005) in men.

**Conclusions:**

A high GLV dietary intervention increased serum zonulin levels and had no effect on fecal zonulin. Lack of concordance between several inflammation-associated biomarkers and zonulin corroborate recent reports of limited utility of zonulin in obese adults free of lower gastrointestinal disease.

*Trial Registration information*: https://clinicaltrials.gov/ct2/show/NCT03582306 (NCT03582306) registered on 07/11/2018.

**Supplementary Information:**

The online version contains supplementary material available at 10.1186/s12876-022-02248-3.

## Introduction

Intestinal permeability is linked to many disease states including autoimmune, hepatic and neurological diseases, diabetes, and irritable bowel syndrome (IBS) [1]. Tight junction permeability in the epithelial tissue of the intestine is regulated through numerous physiological factors [2]. One factor, the protein zonulin, has been proposed to be a major pathophysiological regulator [2, 3]. Zonulin has been shown to be elevated in diabetes [4–6], IBS and irritable bowel disease (IBD) [7–9], Colitis [10, 11], and Celiac disease [12–14]. Zonulin plays a role in these disease states as the gatekeeper of permeability. Though the exact triggers of zonulin upregulation are still under investigation, it is known to open the tight junctions in the intestine and allow for increased bacterial translocation. This translocation into the blood stream causes systemic inflammatory responses that have been found in the previously mentioned disease states. Zonulin receptors have also been found in the brain and in association with neuro-inflammatory diseases. This shows an even greater need to understand the triggers of modulators of this tight junction permeability regulator [15–20].

A potential mechanism by which zonulin leads to tight junction disassembly and intestinal permeability pathogenesis is through a reversible signaling pathway. Zonulin causes the phosphorylation of zonulin occluden-1 complex (ZO1) and myosin 1C, which results in the displacement of ZO1. Subsequent breakdown of the tight junction linkage causes increased intercellular permeability [21]. The increased permeability allows bacterial translocation. As the bacteria and bacteria metabolites enter the blood stream, there is an immune response and increased circulating inflammatory cytokines including C-reactive protein (CRP), interleukin-6 (IL-6), and tumor necrosis factor alpha (TNFα) [22]. This leads to inflammation and further intracellular tight junction breakdown which creates a cycle of greater bacterial translocation and then more inflammation [23]. Since zonulin is a key step in the start of systemic inflammation in this model, understanding triggers of this pathway is key for improving chronic inflammation and the diseases associated with it.

The modulators and purpose of zonulin production within the human population remain unclear. Physiologically, zonulin is theorized to promote the flushing of potentially harmful bacteria and other detrimental molecules out of the small intestine by increasing water entering the GI tract [24]. Therefore, the gut microbiome has become a focal point for contributing factors of zonulin secretion and tight junction permeability. Interestingly, the human analogue of zonulin was discovered after a zonulin occludin toxin was found to be produced by *Vibrio cholera,* which is known to cause severe diarrhea via opening of intestinal tight junctions [25, 26]. The opening of tight junctions with the presence of bacteria allows for possible entrance of these bacteria, and their components, into the blood stream. Lipopolysaccharide (LPS), a structural component of gram-negative bacteria, can break off these bacteria and cause endotoxemia. Thus, LPS binding protein (LBP) serves as surrogate for bacterial translocation [27] that would be followed by acute phase proteins including CRP [28] and alpha-1-acid glycoprotein (ORM-1) [29]. Other bacteria have been associated with changes in zonulin levels, though the mechanism of action has yet to be elucidated.

Diet has been studied in relation to zonulin secretion as well. Gliadin, a protein in wheat, was found to trigger zonulin production in early research on Celiac Disease [13, 30]. Several dietary factors have since been investigated in relation to zonulin, ranging from dietary patterns to individual nutrients. One dietary pattern of interest is the Western diet due to its association with increased zonulin production and intestinal permeability and wide spread consumption [1]. This diet is characterized by a high intake of red and processed meats, low intake of vegetables, whole grains, and other health prudent foods [31]. This suggests improving overall diet quality may be an avenue to minimize zonulin expression and improve gut health.

We recently conducted the Meat and Three Greens (M3G) Feasibility Trial which aimed to reduce colorectal cancer risk via a high green leafy vegetable (GLV) dietary intervention. This intervention was designed to reduce the colonocyte damage associated with heme in red meat by increasing GLVs high in fiber and chlorophyll; intended to reduce colorectal cancer risk. Chlorophyll can bind to heme and reduce the cytotoxic effects while the prebiotic fiber can improve the microbiome which can influence inflammation and the health of the colonocytes [32]. Though zonulin is present only in the small intestine, we hypothesized that this intervention may also decrease zonulin and reduce intestinal permeability. The cytotoxic effects of heme in red meat is not exclusive to the colon and has been shown to be carcinogenic in the small intestine [33]. The reduction of the cytotoxicity of red meat in the small intestine may also impact small intestine permeability through zonulin due to its wide spread association with many disease states. Thus, we sought to determine whether our high GLV intervention reduced serum and fecal zonulin. Additionally, we report data for related biomarkers, diet and microbiota and their associations with zonulin.

## Methods

### Participants

**Fig. 1:**
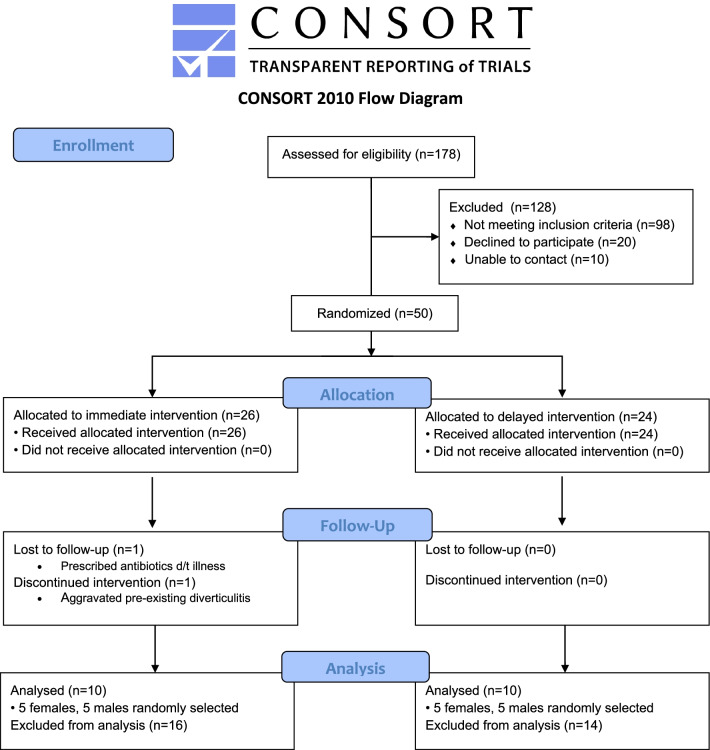
Consort diagram for participants in the M3G feasibility trial included in zonulin analyses

The M3G feasibility trial was conducted from July 2018 to December 2018. Detailed methods have been previously described [32, 34]. Participants were recruited from July to September 2018 and researchers obtained written consent prior to post-screening data collection. Interested individuals were deemed eligible for participation if the following criteria were met: (1) low GLV intake (< 2servings/day); (2) high red meat consumption (> 5 servings/week); (3) BMI categorized as obese (> 30 kg/m^2^); (4) willing to continue current diet patterns throughout study period; (5) willing to adhere to dietary intervention during 4-week period; (6) capability to store and cook supplied frozen vegetables; (7) ability to fluently speak and read English. Exclusion criteria include previous: colorectal cancer diagnosis or use of antibiotic, corticosteroid, immunosuppressive agents, or commercial probiotics. All participants completed the intervention, washout, and control periods, each lasting 4 weeks; randomization assigned participants to intervention-first or control-first groups. This study was approved by the Auburn University Institutional Review Board, protocol # 18-180 EP 1806, and was conducted according to the Declaration of Helsinki. The study was pre-registered on ClinicalTrials.gov (NCT03582306) on 11/07/2018. The aims of the main report were determined a-priori and are included in the ClinicalTrials registry, though this study is a secondary analysis with additional biomarkers analyzed. For this secondary analysis, a random number generated selection of five males and five females with complete biological data from each randomization group of the M3G study were chosen, creating a subset of 20 participants (Fig. 1).

### Intervention

During the intervention period, GLV were provided to participants in the form of frozen spinach, kale, collards, mustard greens, and turnip greens. A recipe book was also provided to facilitate continued preparation and consumption of GLV. Participants were instructed to consume their normal diet during the 4-week washout and control periods.

### Data collection

Dietary recalls were collected for two 24-h periods prior to each visit and entered into the Automated Self-Administered 24-Hour Dietary Assessment tool (ASA24) by a Registered Dietitian or dietetics student [35]. Height and weight were measured using standard procedures and used to determine BMI. Hip and waist circumference were measured using a tape measure. Body composition was determined using a handheld Body Impedance Analysis (BIA) system (Omron HBF-306C, Omron Healthcare, Inc. Lake Forest IL).

Stool samples were collected with a commode specimen collector and sterile collection tubes. Samples were sealed and immediately placed in the participant’s freezer until their next appointment. Fecal water was isolated as previously described [32]. Fecal and serum zonulin levels were determined using a competitive enzyme-linked immunosorbent assay kit (ELISA) (Immunodiagnostik AG, Berlin Germany, Cat. #KR5600.20 and #KR5601) [36, 37]. Fecal microbial DNA was isolated using Zymo Research kits (Irvine, CA, USA, Cat. #D6010). The 250-base pair V4 region of the rRNA gene was amplified by polymerase chain reaction and sequenced using the Illumina Miseq instrument (San Diego, CA, USA) and analyzed within the Quantitative Insight into Microbial Ecology (QIME) suite, version 1.7.

Blood samples were obtained by a trained phlebotomist and separated into serum and plasma before being frozen and stored at -80 °C until analysis. Circulating biomarkers with known associations to various gut microbes and chronic inflammation were quantified to assess inflammation [38]. Objective adherence to study protocol was assessed using changes in plasma Vitamin K1, as GLVs are the primary source of Vitamin K1 [39]. CRP was measured via an ELISA assay from RayBiotech (Peachtree Corner, GA, USA, Cat. #ELH-CRP). IL-6 and TNFα were measured via ELISA assay from ABCam (Cambridge, UK, Cat. #ab178013 and #ab181421). LBP and ORM-1 were measured via kits from R&D Systems (Minneapolis, MN, USA, Cat. #DY870-05 and #DY3694). Vitamin K was analyzed via liquid chromatography–mass spectrometry as previously described [32].

### Statistical analysis

Based on the results reported by Aasbrenn et al., which reported changes in serum zonulin that correlated with changes in gastrointestinal symptoms, a clinically significant 30% reduction in zonulin with a sample size of 19 would result in 0.808 power with an effect size of 0.686 at the 0.05 significance level [40].

Due to the crossover design of the M3G study, both the immediate and delayed groups completed the same 4-week intervention and control periods. Thus, pooled comparisons were made between changes during intervention and control periods. Descriptive statistics were obtained for study participants, and sexes compared using Mann–Whitney U Tests for continuous variables and chi-square tests for categorical variables. Spearman correlations were used to discern the relationships between bacterial taxa and other outcome variables and Bonferroni correction was used to correct for multiple testing of correlations with dietary changes, cytokines, and the microbiome in Additional file 2: Fig. S2 and Additional file 3: Fig. S3. Changes in biomarkers, dietary intake variables and microbial taxa between periods were assessed with Wilcoxon Sign-Rank tests. Statistical significance threshold was set at *p* = 0.05.

## Results

Study Participants: The 20 participants in this study were 50 ± 14 years old and on average had Class II Obesity (36.2 ± 4.6 kg/m^2^). Rates of cardiovascular disease (44%), Type II Diabetes (10%), and gastrointestinal disease (20%, most commonly gastro-esophageal reflux) were generally low. The majority of participants identified as white and had advanced degrees. The female cohort had significantly lower hip to waist ratio and higher body fat percentage (*p* < 0.001 for both) than the male participants, as would be expected given similar BMI. From study enrollment to follow-up, bodyweight (108.9 ± 16.9 kg to 108.4 ± 17.7 kg, *p* = 0.459), waist-to-hip circumference (0.95 ± 0.08 to 0.94 ± 0.07, *p* = 0.876), and body fat percentage (37.7 ± 6.9% to 37.7 ± 6.8%, *p* = 0.989) did not change significantly. Complete demographics of the participants are presented in Table 1.

**Table 1:** Demographic, anthropometric measures of adults (n = 20) participating in a high green leafy vegetable dietary intervention

	All	Female	Male	*p* value
	(n = 20)	(n = 10)	(n = 10)	
Age (years)*	50 (14)	53.4 (14.5)	46.7 (12.8)	0.353
Body Mass Index (kg/m^2^)*	36.2 (4.6)	35.6 (4.3)	36.0 (4.2)	0.912
Waist to hip ratio*	0.95 (0.08)	0.89 (0.04)	1 (0.07)	**< 0.001**
Body fat percentage*	37.7 (7.0)	43.3 (4.8)	32.2 (3.4)	**< 0.001**
RM servings per week*	9.1 (3.4)	8.2 (3.0)	10.0 (3.8)	0.165
GLV servings per week*	0.16 (0.18)	0.2 (0.3)	0.1 (0.1)	0.912
Cardiovascular disease§	9 (45)	4 (40)	5 (50)	1.000
Type II Diabetes§	2 (10)	1 (10)	1 (10)	1.000
Gastrointestinal disease§	4 (20)	2 (20)	2 (20)	1.000
Race§				0.582
African-American	4 (20)	1 (10)	3 (30)	
White	16 (80)	9 (90)	7 (70)	
Education§				0.587
Associate degree or less	4 (20)	2 (20)	2 (20)	
Bachelor's degree	6 (30)	2 (20)	4 (40)	
Advanced degree(s)	10 (50)	6 (60)	4 (40)	
Marital Status§				0.650
Married	8 (40)	3 (30)	5 (50)	
Not currently Married	12 (60)	7 (70)	5 (50)	

Demographic and anthropometric measures were taken from adults participating in a high green leafy vegetable dietary intervention

^*^Values displayed as Mean (SD)

^§^values displayed as N (%); Bolded p-values show significant differences (*p* < 0.05)

### Biomarkers

In all participants, serum zonulin increased and fecal zonulin was relatively unchanged in the intervention compared to control periods (Table 2). Plasma vitamin K1 increased while plasma 8OHdG decreased in the intervention compared to the control period for all participants. Significant changes in ORM1 within the female group drove the whole group significance for that biomarker (*p* = 0.020) and only females had a larger increase in ORM-1 during the control period compared to the intervention period. Additionally, CRP increased in females following the intervention. (Additional file 4: Table S1).

**Table 2:** Biomarkers of adults (n = 20) participating in a high green leafy vegetable dietary intervention

	Baseline	Intervention Change	Control Change	*p* value
	Mean (SD)			
Zonulin (ng/mL)	4.13 (0.55)	0.16 (0.62)	− 0.33 (1.07)	0.108
Fecal Zonulin (ng/mL)	4.94 (4.91)	− 1.3 (5.39)	− 1.56 (4.62)	0.575
LBP (ng/mL)	3.62 (2.65)	− 0.49 (2.87)	1.15 (4.82)	0.279
ORM-1 (pg/mL)	1117.75 (1001.2)	26.91 (1163.12)	127.43 (1049.33)	0.601
Vitamin K1 (ng/mL)	0.09 (0.26)	0.65 (1.02)	0.03 (0.32)	**0.005**
8OHdG (ng/mL)	39.67 (23.59)	− 8.84 (17.08)	0.84 (7.1)	**0.023**
Fecal 8OHdG (µg/mL)	27.56 (73.41)	− 11.00 (47.25)	− 10.93 (38.62)	0.627
TNFα (pg/mL)	154.41 (43.41)	− 25.27 (48.46)	− 0.67 (35.69)	0.247
IL6 (pg/mL)	5.57 (3.42)	1.41 (3.66)	0.83 (6.9)	0.575
CRP (ng/mL)	3.961 (4.77255)	− 0.5881 (5.08918)	− 0.9856 (4.6389)	0.601

Bold values indicate significant differences between changes during intervention period versus control period

**Fig. 2:**
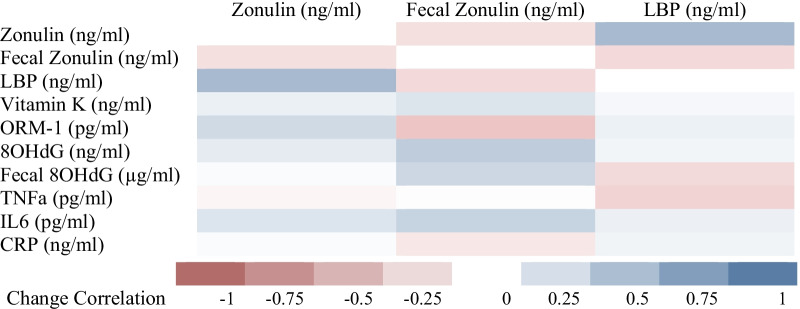
Correlations between changes in biomarkers and IP markers during a high GLV diet

To understand the relationship of between inflammatory markers and intestinal permeability following a high GLV intervention, we examined associations of circulating cytokines and acute-phase proteins with IP-related biomarkers, serum zonulin, fecal zonulin, and serum LBP. At baseline, there were no significant correlations between zonulin and other biomarkers among all participants. Similarly, no relationships were observed when changes in zonulin and other biomarkers were assessed (Fig. 2). In the female group, inverse relationships between TNFa and both LBP and zonulin were observed (*p* < 0.001, *p* = 0.011 respectively). Although not significant, most other biomarkers within the female group had positive correlations with the zonulin, fecal zonulin, and LBP. In the male group, none of the correlations reached statistical significance (Additional file 1: Fig. S1).

### Description

Heat map of correlations between change in biological markers and zonulin, fecal zonulin, and LBP during the 4-week dietary intervention of high green leafy vegetable intake. Shade of the color indicates strength of correlations, with red indicating negative correlations and blue indicating positive correlations. Significant correlation coefficients indicated with ** (*p* < 0.005).

### Diet

Baseline and study period dietary data are reported in Table 3. There were significantly greater increases in vitamin K and cups of dark GLV intake during the intervention compared to the control period for all participants, indicating adherence to intervention protocol. Within sexes, females increased Vitamin B12 and Vitamin K, while males increased Vitamin K, dark GLV, and whole grains (Additional file 5: Table S2).

**Table 3:** Select nutrient values of obese adults (n = 20) participating in a high green leafy vegetable dietary intervention

All participants	Baseline	Intervention Change	Control Change	p-value2
	Mean (SD)			
Energy (kcal)	2066.07 (591.4)	− 33.62 (985.72)	106.94 (1103.85)	0.970
Protein (g)	83.5 (25.76)	4.68 (38.73)	0.52 (44.96)	0.823
Fat (g)	87.42 (28.34)	1.76 (57.45)	1.11 (47.44)	0.881
Carb (g)	239.9 (78.69)	− 18.13 (110)	23.68 (139.36)	0.526
Sugar (g)	103.38 (48.91)	− 0.66 (56.11)	25.14 (90.76)	0.455
Fiber (g)	16.58 (8.01)	0.95 (8.85)	0.29 (7.35)	0.709
Vitamin B12 (mcg)	7.04 (7)	0.81 (3.36)	1.13 (8.24)	0.232
Vitamin A (mcg)	933.14 (781.98)	433.50 (896.18)	− 91.37 (543.29)	0.023
Vitamin K (mcg)	132.99 (118.81)	596.22 (581.43)	− 22.56 (131.5)	**< 0.001**
α-linoleic acid (g)	2.04 (1.36)	− 0.08 (1.69)	− 0.01 (1.03)	0.794
EPA (g)	0.01 (0.02)	0 (0.05)	0.01 (0.02)	0.264
DHA (g)	0.03 (0.03)	− 0.01 (0.1)	0.02 (0.06)	0.038
Dark GLV (cup)	0.16 (0.24)	0.74 (0.68)	− 0.02 (0.34)	**< 0.001**
Whole grains (oz)	0.9 (0.92)	− 0.36 (0.86)	− 0.03 (0.72)	0.246
Refined grains (oz)	5.17 (1.99)	0.01 (3.99)	− 0.24 (3.93)	0.654
Meat total (oz)	4.78 (2.94)	0.52 (3.52)	− 0.01 (3.45)	0.478
Red meat (oz)	1.8 (1.94)	1.04 (2.39)	− 0.3 (1.57)	0.073
Cured meat (oz)	1.03 (1.23)	− 0.33 (1.41)	− 0.16 (2.11)	0.526

Bold values indicate significant differences between changes during intervention period versus control period

No significant associations were observed between dietary variables and biomarkers after correcting for multiple comparisons; however, negative correlations with serum zonulin and most macronutrients almost reached significance in females (Additional file 2: Fig. S2). Additionally, inverse correlations between plasma 8OhdG and dietary polyunsaturated fatty acids were observed in females. Serum IL-6 had the strongest negative association with α-linolenic acid in females, however the relationship was positive for males. In males, serum zonulin was weakly but positively correlated with most of the reported dietary components (Additional file 2: Fig. S2).

### Microbiome

**Fig. 3:**
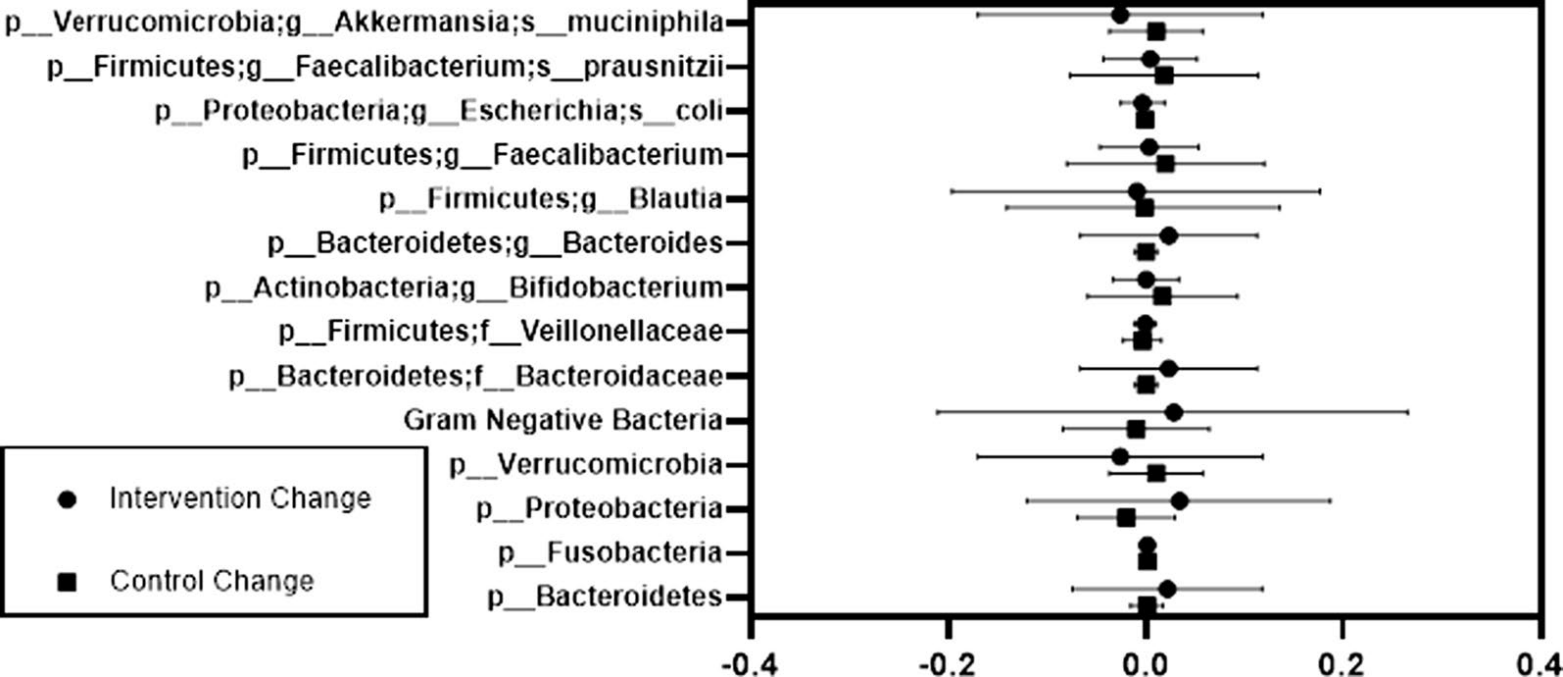
Changes in bacterial taxa associated with Zonulin during the intervention and control periods of a crossover high green leafy vegetable dietary intervention

The gut microbiome greatly influences the health and permeability of the intestine via inflammatory and other pathways, thus we investigated specific bacteria known to interact with these signals [41]. There were no statistically significant differences in change of the microbiome between the intervention and control periods (Fig. 3). In females, the bacterial phylum Proteobacteria was negatively correlated with serum zonulin (*p* = 0.001) and positively correlated with TNFα (*p* = 0.002). Fusobacteria and CRP were also positively correlated in females (*p* = 0.002). In males, serum zonulin correlated negatively with the genus *Bifidobacterium* (*p* = 0.009), and positively with the genus *Bacteroides* (*p* = 0.005). *Faecalibacterium* and *Faecalibacterium prausnitzii* were inversely associated with fecal 8OHdG (*p* = 0.001 for both) (Additional file 3: Fig. S3).

## Discussion

Herein, we sought to explore the effects and routes of a high GLV intervention on IP-related biomarkers. Thus, we have fully described dietary, biological, and microbiota changes in conjunction with these IP-related proteins in blood and fecal samples in this longitudinal study. Utilizing these variables, we were able to explore potential zonulin modulators. Contrary to our hypothesis, a high GLV dietary intervention had null effects on zonulin and LBP. In our sample population, there were expected anthropometric differences between males and females, but all had a BMI greater than 30 kg/m^2^ as required for study participation. Both groups maintained their anthropometric measures during the study and all participants were similar ages, removing these as potential confounding variables in our study [42, 43].

The inclusion of GLV in the diet provided a source of three proposed zonulin modulators: vitamin A, vitamin K, and fiber [22]. There is limited evidence to suggest that Vitamin A and K play a role in zonulin production so far. Xiao et al. investigated vitamin A deficient mice fed a vitamin A supplement and observed a negative association between serum zonulin and vitamin A levels [44]. In an observational study of pregnant overweight women, a negative relationship between serum zonulin and vitamin A and K levels was observed, though most participants’ vitamin A and K consumption was below recommended levels [45]. Our study participants consumed adequate levels of both vitamins, which indicates vitamins A and K may influence zonulin levels only when a deficiency is present.

Interestingly, various studies have associated fiber with zonulin through dietary interventions with whole foods high in fiber and as fortification into a meal [45–47]. Despite the addition of GLV into the diet, fiber intake was not significantly increased during the intervention period; therefore, any relationship between fiber and zonulin was challenging to discern. Alternatively, vitamin B12 has been positively correlated with zonulin [37], and we did observe significant increases in Vitamin B12 consumption within our female participants. The lack of correlation to zonulin may have been caused by their baseline consumption exceeding the threshold of intake suggested by this aforementioned study. Furthermore, our male participants showed more consistent Vitamin B12 consumption from intervention to control period.

Several other dietary factors have been correlated with either circulating or fecal zonulin including: wheat/gliadin [13, 30], total calories [37], total protein [37, 45, 48], total carbohydrates [37, 48], fat percentage in diet [43], Omega-3 PUFA [45]. None of these other dietary components were significantly changed during this intervention which limits the potential for finding significant relationships. Though single nutrients have been shown to effect zonulin levels in many observational and mouse studies, human dietary interventions have had trouble replicating correlations with the increase of single nutrients. Some studies have suggested BMI, waist circumference, and aging may have stronger correlations to zonulin than dietary changes [42, 48–50].

Alterations in intestinal permeability are associated with systemic inflammation and are observed in many immune-mediated disease states [51]. With increased intestinal permeability, greater number of bacteria can translocate into the bloodstream. LBP was significantly decreased during the intervention period despite the significant increase in zonulin. Zonulin, released in response to known factors like gliadin or certain bacteria, results in an immune response and subsequent inflammation [24]. Within obese and diseased populations, various studies indicate zonulin is positively associated with both intestinal permeability and inflammatory cytokines [22, 37, 43]. Alternatively, our study found LBP had an inverse relationship with both the inflammatory marker TNFα and serum zonulin within the female group, although the latter did not reach statistical significance. Finally, the male group had very weak correlations between biomarkers.

It is well-established that the gut microbiome plays a role in intestinal health and permeability. Furthermore, zonulin has been associated with different phylogenetic levels of various bacteria, dysbiosis, small intestine bacterial overgrowth, and probiotic supplementation [36, 43, 45, 52–54]. Herein, we found Proteobacteria, which can contribute to dysbiosis and promote intestinal inflammation [55], to be positively correlated with zonulin in the female group. The male group had multiple relationships that were strong, but not significant, including Bacteroidaceae family, *Bifidobacterium*, and *Escherichia coli*, as well as total gram-negative bacteria. Bacteroidaceae, *E. Coli*, and other gram-negative bacteria are associated with zonulin secretion and lipopolysaccharide-induced inflammatory responses [45, 53], although the direct mechanism remains unknown. *Bifidobacterium*, on the other hand, has been shown to have a negative correlation with zonulin and may have a protective effect on intestinal permeability, indicated in cell cultures exposed to lipopolysaccharide [54], although this should be explored in humans.

### Limitations

Though our hypothesis was not supported by this exploratory study, we have reported numerous outcomes and relationships between biological and dietary factors in relation to zonulin and other biomarkers. Nonetheless, several limitations are noted. First, the intervention was not designed with zonulin as a primary aim and similarly was not powered to detect differences or control for confounding variables. The small sample size and uniqueness of our sample limits the generalizability of our findings. Our sample was limited to adults with BMI > 30 kg/m^2^ with high habitual red meat intake. Also, our sample had a lower percentage of African American individuals (20%) than the Alabama state average (26.8%) and higher percentage than the national average (13.4%) [56, 57]. Due to recruiting from university faculty and staff, a higher percentage of individuals had a Bachelor’s degree or higher (80%) than the state average (24.9%) and the national average (31.5%) [56, 57]. Also noteworthy is that the ELISA designed for zonulin has been shown to bind to other molecules related to zonulin and could potentially alter results [58]. Nonetheless, this is still the best method for measuring zonulin in human samples and widely used for measuring intestinal permeability.

## Conclusion

A high green leafy vegetable dietary intervention aimed to reduce colon cancer risk increased serum zonulin in adults with obesity, contrary to our hypothesis. Lack of concordance between several inflammation-associated biomarkers and zonulin corroborate recent reports of limited utility of zonulin in obese adults free of lower gastrointestinal disease.

## Supplementary Information

**Additional file 1: Fig. S1**. Sex specific change correlations between changes in biomarkers and IP markers during a high GLV diet.

**Additional file 2: Fig. S2**. Sex specific change correlations between biomarkers and nutrients during a high GLV diet.

**Additional file 3: Fig. S3**. Sex specific change correlations between change in fecal bacteria and biological markers during a high GLV diet.

**Additional file 4: Table S1**. Biomarkers of each sex (n=10 for both) participating in a high green leafy vegetable dietary intervention.

**Additional file 5: Table S2**. Select nutrient values of each sex (n=10 for both) participating in a high green leafy vegetable dietary intervention.

## Data Availability

The datasets used and/or analyzed during the current study are available from the corresponding author on reasonable request.

## Abbreviations

IBS: Irritable bowel syndrome
IBD: Irritable bowel disease
ZO1: Zonulin occluden-1 complex
CRP: C-reactive protein
IL-6: Interleukin-6
TNFα: Tumor necrosis factor alpha
M3G: Meat and Three Greens feasibility trial
GLV: Green leafy vegetables
BMI: Body mass index
LBP: Lipopolysaccharide binding protein
ORM1: Alpha-1-acid glycoprotein

## References

[1] Leech B, Schloss J, Steel A (2019). Association between increased intestinal permeability and disease: a systematic review. Adv Integr Med.

[2] Graziani C (2019). Intestinal permeability in physiological and pathological conditions: major determinants and assessment modalities. Eur Rev Med Pharmacol Sci.

[3] Wang W (2000). Human zonulin, a potential modulator of intestinal tight junctions. J Cell Sci.

[4] Jayashree B (2014). Increased circulatory levels of lipopolysaccharide (LPS) and zonulin signify novel biomarkers of proinflammation in patients with type 2 diabetes. Mol Cell Biochem.

[5] Moreno-Navarrete JM (2012). Circulating zonulin, a marker of intestinal permeability, is increased in association with obesity-associated insulin resistance. PLoS ONE.

[6] Sapone A (2006). Zonulin upregulation is associated with increased gut permeability in subjects with type 1 diabetes and their relatives. Diabetes.

[7] Singh, P., et al., Serum zonulin is elevated in IBS and correlates with stool frequency in IBS-D. United Eur. Gastroenterol. J. 2019:2050640619826419. 10.1177/2050640619826419 PMC6545708 31210949

[8] Caviglia GP (2019). Serum zonulin in patients with inflammatory bowel disease: a pilot study. Minerva Med.

[9] Malíčková K (2017). Fecal zonulin is elevated in Crohn's disease and in cigarette smokers. Pract Lab Med.

[10] Sturgeon C, Lan J, Fasano A (2017). Zonulin transgenic mice show altered gut permeability and increased morbidity/mortality in the DSS colitis model. Ann N Y Acad Sci.

[11] Arrieta MC (2008). Reducing small intestinal permeability attenuates colitis in the IL10 gene-deficient mouse. Gut.

[12] Rauhavirta T (2014). Impaired epithelial integrity in the duodenal mucosa in early stages of celiac disease. Transl Res.

[13] Drago S (2006). Gliadin, zonulin and gut permeability: Effects on celiac and non-celiac intestinal mucosa and intestinal cell lines. Scand J Gastroenterol.

[14] Fasano A (2000). Zonulin, a newly discovered modulator of intestinal permeability, and its expression in coeliac disease. The Lancet.

[15] Zhang D (2015). Serum zonulin is elevated in women with polycystic ovary syndrome and correlates with insulin resistance and severity of anovulation. Eur J Endocrinol.

[16] Lindheim L (2017). Alterations in gut microbiome composition and barrier function are associated with reproductive and metabolic defects in women with polycystic ovary syndrome (PCOS): a pilot study. PLoS ONE.

[17] Esnafoglu E (2017). Increased serum zonulin levels as an intestinal permeability marker in autistic subjects. J Pediatr.

[18] Schwiertz A (2018). Fecal markers of intestinal inflammation and intestinal permeability are elevated in Parkinson's disease. Parkinsonism Relat Disord.

[19] Özyurt G (2018). Increased zonulin is associated with hyperactivity and social dysfunctions in children with attention deficit hyperactivity disorder. Compr Psychiatry.

[20] Rahman MT (2018). IFN-γ, IL-17A, or zonulin rapidly increase the permeability of the blood-brain and small intestinal epithelial barriers: Relevance for neuro-inflammatory diseases. Biochem Biophys Res Commun.

[21] Fasano A (2000). Regulation of intercellular tight junctions by zonula occludens toxin and its eukaryotic analogue Zonulin. Ann N Y Acad Sci.

[22] Leech B (2019). Risk factors associated with intestinal permeability in an adult population: a systematic review. Int J Clin Pract.

[23] Sturgeon C, Fasano A (2016). Zonulin, a regulator of epithelial and endothelial barrier functions, and its involvement in chronic inflammatory diseases. Tissue Barriers.

[24] Fasano A (2011). Zonulin and its regulation of intestinal barrier function: the biological door to inflammation, autoimmunity, and cancer. Physiol Rev.

[25] Fasano A (1997). The enterotoxic effect of zonula occludens toxin on rabbit small intestine involves the paracellular pathway. Gastroenterology.

[26] Fasano A (1991). Vibrio cholerae produces a second enterotoxin, which affects intestinal tight junctions. Proc Natl Acad Sci.

[27] Zhao S (2017). Akkermansia muciniphila improves metabolic profiles by reducing inflammation in chow diet-fed mice. J Mol Endocrinol.

[28] Mold C (2002). C-reactive protein mediates protection from lipopolysaccharide through interactions with FcγR. J Immunol.

[29] Moore DF (1997). Alpha-1-acid (AAG, orosomucoid) glycoprotein: interaction with bacterial lipopolysaccharide and protection from sepsis. Inflammation.

[30] Clemente MG (2003). Early effects of gliadin on enterocyte intracellular signalling involved in intestinal barrier function. Gut.

[31] Hu FB (1999). Reproducibility and validity of dietary patterns assessed with a food-frequency questionnaire. Am J Clin Nutr.

[32] Frugé AD (2021). A dietary intervention high in green leafy vegetables reduces oxidative DNA damage in adults at increased risk of colorectal cancer: biological outcomes of the randomized controlled meat and three greens (M3G) feasibility trial. Nutrients.

[33] Pan SY, Morrison H (2011). Epidemiology of cancer of the small intestine. World J Gastrointest Oncol.

[34] Frugé AD (2019). Primary outcomes of a randomized controlled crossover trial to explore the effects of a high chlorophyll dietary intervention to reduce colon cancer risk in adults: the meat and three greens (M3G) feasibility trial. Nutrients.

[35] Subar AF (2012). The Automated Self-Administered 24-hour dietary recall (ASA24): a resource for researchers, clinicians, and educators from the National Cancer Institute. J Acad Nutr Diet.

[36] Lamprecht M (2012). Probiotic supplementation affects markers of intestinal barrier, oxidation, and inflammation in trained men; a randomized, double-blinded, placebo-controlled trial. J Int Soc Sports Nutr.

[37] Mörkl S (2018). Gut microbiota, dietary intakes and intestinal permeability reflected by serum zonulin in women. Eur J Nutr.

[38] Xiao S (2014). A gut microbiota-targeted dietary intervention for amelioration of chronic inflammation underlying metabolic syndrome. FEMS Microbiol Ecol.

[39] Booth SL, Suttie JW (1998). Dietary intake and adequacy of vitamin K. J Nutr.

[40] Aasbrenn M, Lydersen S, Farup PG (2020). Changes in serum zonulin in individuals with morbid obesity after weight-loss interventions: a prospective cohort study. BMC Endocr Disord.

[41] Teixeira TFS (2012). Potential mechanisms for the emerging link between obesity and increased intestinal permeability. Nutr Res.

[42] Qi Y (2017). Intestinal permeability biomarker zonulin is elevated in healthy aging. J Am Med Dir Assoc.

[43] Zak-Gołąb A (2013). Gut microbiota, microinflammation, metabolic profile, and zonulin concentration in obese and normal weight subjects. Int J Endocrinol.

[44] Xiao L (2019). Vitamin A supplementation improves the intestinal mucosal barrier and facilitates the expression of tight junction proteins in rats with diarrhea. Nutrition.

[45] Mokkala K (2016). Gut microbiota richness and composition and dietary intake of overweight pregnant women are related to serum Zonulin concentration, a marker for intestinal permeability. J Nutr.

[46] Krawczyk M (2018). Gut permeability might be improved by dietary fiber in individuals with nonalcoholic fatty liver disease (NAFLD) undergoing weight reduction. Nutrients.

[47] Russo F (2012). Inulin-enriched pasta improves intestinal permeability and modifies the circulating levels of zonulin and glucagon-like peptide 2 in healthy young volunteers. Nutr Res.

[48] Ohlsson B (2017). Calprotectin in serum and zonulin in serum and feces are elevated after introduction of a diet with lower carbohydrate content and higher fiber, fat and protein contents. Biomedical reports.

[49] Ohlsson B (2019). An Okinawan-based Nordic diet improves glucose and lipid metabolism in health and type 2 diabetes, in alignment with changes in the endocrine profile, whereas zonulin levels are elevated. Exp Ther Med.

[50] Ohlsson B, Orho-Melander M, Nilsson PM (2017). Higher levels of serum Zonulin may rather be associated with increased risk of obesity and hyperlipidemia, than with gastrointestinal symptoms or disease manifestations. Int J Mol Sci.

[51] Arrieta MC, Bistritz L, Meddings JB (2006). Alterations in intestinal permeability. Gut.

[52] Giorgi A (2020). A probiotic preparation hydrolyzes gliadin and protects intestinal cells from the toxicity of pro-inflammatory peptides. Nutrients.

[53] Asmar RE (2002). Host-dependent zonulin secretion causes the impairment of the small intestine barrier function after bacterial exposure. Gastroenterology.

[54] Ling X (2016). Protective effects of bifidobacterium on intestinal barrier function in LPS-induced enterocyte barrier injury of Caco-2 monolayers and in a rat NEC model. PLoS ONE.

[55] Carvalho FA (2012). Transient inability to manage proteobacteria promotes chronic gut inflammation in TLR5-deficient mice. Cell Host Microbe.

[56] Bureau., U.S.C., QuickFacts Alabama. 2020: https://www.census.gov/quickfacts/AL.

[57] Bureau., U.S.C., Quickfacts United States. 2020: https://www.census.gov/quickfacts/fact/table/US/PST045218.

[58] Scheffler L (2018). Widely Used Commercial ELISA Does Not Detect Precursor of Haptoglobin2, but Recognizes Properdin as a Potential Second Member of the Zonulin Family. Front Endocrinol (Lausanne).

