# Supplementary material for: Plasma and fecal zonulin are not altered by a high green leafy vegetable dietary intervention: secondary analysis of a randomized control crossover trial
Source: BMC Gastroenterol. 2022 Apr 12;22:184. doi: 10.1186/s12876-022-02248-3 (PMC9004007; doi:10.1186/s12876-022-02248-3)
Supplement: Supplementary file 1 — Additional file 1: Fig. S1. Sex specific change correlations between changes in biomarkers and IP markers during a high GLV diet. [file 12876_2022_2248_MOESM1_ESM.docx]

Additional file 1: Fig. S1. Sex specific change correlations between changes in biomarkers and IP markers during a high GLV diet

|  | Female | | | | | | |  | Male | | | | | | |
| --- | --- | --- | --- | --- | --- | --- | --- | --- | --- | --- | --- | --- | --- | --- | --- |
|  | Zonulin (ng/ml) | | Fecal Zonulin (ng/ml) | | LBP (ng/ml) | | |  | Zonulin (ng/ml) | | | Fecal Zonulin (ng/ml) | | LBP (ng/ml) | |
| Zonulin (ng/ml) |  | |  | |  | | |  |  | | |  | |  | |
| Fecal Zonulin (ng/ml) |  | |  | |  | | |  |  | | |  | |  | |
| LBP (ng/ml) |  | |  | |  | | |  |  | | |  | |  | |
| Vitamin K (ng/ml) |  | |  | |  | | |  |  | | |  | |  | |
| ORM-1 (pg/ml) |  | |  | |  | | |  |  | | |  | |  | |
| 8OHdG (ng/ml) |  | |  | |  | | |  |  | | |  | |  | |
| Fecal 8OHdG (µg/ml) |  | |  | |  | | |  |  | | |  | |  | |
| TNFa (pg/ml) |  | |  | |  | | |  |  | | |  | |  | |
| IL6 (pg/ml) |  | |  | |  | | |  |  | | |  | |  | |
| CRP (ng/ml) |  | |  | |  | | |  |  | | |  | |  | |
|  |  |  | |  | |  |  | | |  |  | |  | |  |
|  |  |  | |  | |  |  | | |  |  | |  | |  |
| Change Correlation | -1 | -0.75 | | -0.5 | | -0.25 | 0 | | | 0.25 | 0.5 | | 0.75 | | 1 |

Description: Heat map of correlations between change in biological markers and zonulin, fecal zonulin, and LBP during the 4-week dietary intervention of high green leafy vegetable intake. Shade of the color indicates strength of correlations, with red indicating negative correlations and blue indicating positive correlations. Significant correlation coefficients are indicated with ** (p<0.005).
