# Supplementary material for: Plasma and fecal zonulin are not altered by a high green leafy vegetable dietary intervention: secondary analysis of a randomized control crossover trial
Source: BMC Gastroenterol. 2022 Apr 12;22:184. doi: 10.1186/s12876-022-02248-3 (PMC9004007; doi:10.1186/s12876-022-02248-3)
Supplement: Supplementary file 2 — Additional file 2: Fig. S2. Sex specific change correlations between biomarkers and nutrients during a high GLV diet. [file 12876_2022_2248_MOESM2_ESM.docx]

Additional file 2: Fig. S2. Sex specific change correlations between biomarkers and nutrients during a high GLV diet

|  |  | Zonulin (ng/ml) | Fecal Zonulin (ng/ml) | | LBP (ng/ml) | | Vitamin K1 (ng/ml) | | ORM1 (pg/ml) | | 8OHdG (ng/mL) | | Fecal 8OHdG (µg/mL) | | TNFa (pg/mL) | | IL6 (pg/mL) | | CRP (ng/mL) | |
| --- | --- | --- | --- | --- | --- | --- | --- | --- | --- | --- | --- | --- | --- | --- | --- | --- | --- | --- | --- | --- |
| **Female** | Energy (kcal) |  |  | |  | |  | |  | |  | |  | |  | |  | |  | |
|  | Protein (g) |  |  | |  | |  | |  | |  | |  | |  | |  | |  | |
|  | Fat (g) |  |  | |  | |  | |  | |  | |  | |  | |  | |  | |
|  | Carb (g) |  |  | |  | |  | |  | |  | |  | |  | |  | |  | |
|  | Sugar (g) |  |  | |  | |  | |  | |  | |  | |  | |  | |  | |
|  | Fiber (g) |  |  | |  | |  | |  | |  | |  | |  | |  | |  | |
|  | Vitamin B12 (mcg) |  |  | |  | |  | |  | |  | |  | |  | |  | |  | |
|  | Vitamin K (mcg) |  |  | |  | |  | |  | |  | |  | |  | |  | |  | |
|  | α-linoleic acid (g) |  |  | |  | |  | |  | |  | |  | |  | |  | |  | |
|  | EPA (g) |  |  | |  | |  | |  | |  | |  | |  | |  | |  | |
|  | DHA (g) |  |  | |  | |  | |  | |  | |  | |  | |  | |  | |
|  | Dark GLV (cup) |  |  | |  | |  | |  | |  | |  | |  | |  | |  | |
|  | Whole grains (oz) |  |  | |  | |  | |  | |  | |  | |  | |  | |  | |
|  | Refined grains (oz) |  |  | |  | |  | |  | |  | |  | |  | |  | |  | |
|  | Meat total (oz) |  |  | |  | |  | |  | |  | |  | |  | |  | |  | |
|  | Red meat (oz) |  |  | |  | |  | |  | |  | |  | |  | |  | |  | |
|  | Cured meat (oz) |  |  | |  | |  | |  | |  | |  | |  | |  | |  | |
|  |  |  |  | |  | |  | |  | |  | |  | |  | |  | |  | |
| **Male** | Energy (kcal) |  |  | |  | |  | |  | |  | |  | |  | |  | |  | |
|  | Protein (g) |  |  | |  | |  | |  | |  | |  | |  | |  | |  | |
|  | Fat (g) |  |  | |  | |  | |  | |  | |  | |  | |  | |  | |
|  | Carb (g) |  |  | |  | |  | |  | |  | |  | |  | |  | |  | |
|  | Sugar (g) |  |  | |  | |  | |  | |  | |  | |  | |  | |  | |
|  | Fiber (g) |  |  | |  | |  | |  | |  | |  | |  | |  | |  | |
|  | Vitamin B12 (mcg) |  |  | |  | |  | |  | |  | |  | |  | |  | |  | |
|  | Vitamin K (mcg) |  |  | |  | |  | |  | |  | |  | |  | |  | |  | |
|  | α-linoleic acid (g) |  |  | |  | |  | |  | |  | |  | |  | |  | |  | |
|  | EPA (g) |  |  | |  | |  | |  | |  | |  | |  | |  | |  | |
|  | DHA (g) |  |  | |  | |  | |  | |  | |  | |  | |  | |  | |
|  | Dark GLV (cup) |  |  | |  | |  | |  | |  | |  | |  | |  | |  | |
|  | Whole grains (oz) |  |  | |  | |  | |  | |  | |  | |  | |  | |  | |
|  | Refined grains (oz) |  |  | |  | |  | |  | |  | |  | |  | |  | |  | |
|  | Meat total oz) |  |  | |  | |  | |  | |  | |  | |  | |  | |  | |
|  | Red meat (oz) |  |  | |  | |  | |  | |  | |  | |  | |  | |  | |
|  | Cured meat (oz) |  |  | |  | |  | |  | |  | |  | |  | |  | |  | |
|  |  |  |  | |  | |  | |  | |  | |  | |  | |  | |  | |
|  |  |  | |  | |  | |  | |  | |  | |  | |  | |  | |  |
|  | Change Correlations | -1 | | -0.75 | | -0.5 | | -0.25 | | 0 | | 0.25 | | 0.5 | | 0.75 | | 1 | |  |

Description: Heat map of correlations between change in biological markers and zonulin, fecal zonulin, and LBP during the 4-week dietary intervention of high green leafy vegetable intake. Shade of the color indicates strength of correlations, with red indicating negative correlations and blue indicating positive correlations. Significant correlation coefficients are indicated with ** (p<0.005).
