# Supplementary material for: Plasma and fecal zonulin are not altered by a high green leafy vegetable dietary intervention: secondary analysis of a randomized control crossover trial
Source: BMC Gastroenterol. 2022 Apr 12;22:184. doi: 10.1186/s12876-022-02248-3 (PMC9004007; doi:10.1186/s12876-022-02248-3)
Supplement: Supplementary file 3 — Additional file 3: Fig. S3. Sex specific change correlations between change in fecal bacteria and biological markers during a high GLV diet. [file 12876_2022_2248_MOESM3_ESM.docx]

|  |  |  | Zonulin (ng/ml) | Fecal Zonulin (ng/ml) | LBP (ng/ml) | ORM-1 (pg/ml) | 8OHdG (ng/mL) | Fecal 8OHdG (µg/mL) | TNFa (pg/mL) | IL6 (pg/mL) | CRP (ng/mL) |
| --- | --- | --- | --- | --- | --- | --- | --- | --- | --- | --- | --- |
| **Female** | Phylum | Bacteroidetes |  |  |  |  |  |  |  |  |  |
|  |  | Fusobacteria |  |  |  |  |  |  |  |  |  |
|  |  | Proteobacteria |  |  |  |  |  |  |  |  |  |
|  |  | Verrucomicrobia |  |  |  |  |  |  |  |  |  |
|  |  | Total gram-negative bacteria |  |  |  |  |  |  |  |  |  |
|  | Class | Bacteroidaceae |  |  |  |  |  |  |  |  |  |
|  |  | Veillonellaceae |  |  |  |  |  |  |  |  |  |
|  | Genus | *Bifidobacterium* |  |  |  |  |  |  |  |  |  |
|  |  | *Bacteroides* |  |  |  |  |  |  |  |  |  |
|  |  | *Blautia* |  |  |  |  |  |  |  |  |  |
|  |  | *Faecalibacterium* |  |  |  |  |  |  |  |  |  |
|  |  | *Escherichia coli* |  |  |  |  |  |  |  |  |  |
|  | Species | *Faecalibacterium prausnitzii* |  |  |  |  |  |  |  |  |  |
|  |  | *Akkermansia muciniphila* |  |  |  |  |  |  |  |  |  |
|  |  |  |  |  |  |  |  |  |  |  |  |
| **Male** | Phylum | Bacteroidetes |  |  |  |  |  |  |  |  |  |
|  |  | Fusobacteria |  |  |  |  |  |  |  |  |  |
|  |  | Proteobacteria |  |  |  |  |  |  |  |  |  |
|  |  | Verrucomicrobia |  |  |  |  |  |  |  |  |  |
|  |  | Total gram-negative bacteria |  |  |  |  |  |  |  |  |  |
|  | Class | Bacteroidaceae |  |  |  |  |  |  |  |  |  |
|  |  | Veillonellaceae |  |  |  |  |  |  |  |  |  |
|  | Genus | *Bifidobacterium* |  |  |  |  |  |  |  |  |  |
|  |  | *Bacteroides* |  |  |  |  |  |  |  |  |  |
|  |  | *Blautia* |  |  |  |  |  |  |  |  |  |
|  |  | *Faecalibacterium* |  |  |  |  |  | ****** |  |  |  |
|  |  | *Escherichia coli* |  |  |  |  |  |  |  |  |  |
|  | Species | *Faecalibacterium prausnitzii* |  |  |  |  |  | ****** |  |  |  |
|  |  | *Akkermansia muciniphila* |  |  |  |  |  |  |  |  |  |
|  |  |  |  |  |  |  |  |  |  |  |  |
|  |  |  |  |  |  |  |  |  |  |  |  |
|  |  |  | -1 | -0.75 | -0.5 | -0.25 | 0 | 0.25 | 0.5 | 0.75 | 1 |

Additional file 3: Fig. S3. Supplemental Figure 3. Sex specific change correlations between change in fecal bacteria and biological markers during a high GLV diet.

Description: Heat map of correlations between change in fecal phyla and select operational taxonomic units and biological markers during the intervention period of a high green leafy vegetable dietary intervention. Shade of the color shows strength of correlations with red indicating negative correlations and blue indicating positive correlations. Significant correlation coefficients are indicated with ** (p<0.0021).
