# Supplementary material for: Plasma and fecal zonulin are not altered by a high green leafy vegetable dietary intervention: secondary analysis of a randomized control crossover trial
Source: BMC Gastroenterol. 2022 Apr 12;22:184. doi: 10.1186/s12876-022-02248-3 (PMC9004007; doi:10.1186/s12876-022-02248-3)
Supplement: Supplementary file 4 — Additional file 4: Table S1. Biomarkers of each sex (n=10 for both) participating in a high green leafy vegetable dietary intervention. [file 12876_2022_2248_MOESM4_ESM.docx]

|  | Baseline | Intervention Change | Control Change |  |
| --- | --- | --- | --- | --- |
| Females (n=10) | -------- Mean (SD) -------- | | | p-value |
| Zonulin (ng/mL) | 4.2 (0.49) | 0.28 (0.6) | -0.4 (1.24) | 0.004 |
| Fecal Zonulin (ng/mL) | 4.01 (5.1) | -1.94 (5.32) | -2.31 (5.31) | 0.239 |
| LBP (ng/mL) | 2.6 (1.52) | 0.03 (1.77) | 2.66 (6.55) | 0.141 |
| ORM-1 (pg/mL) | 1014.23 (810.72) | 463.03 (1054.11) | 176.6 (1347.85) | 0.020 |
| Vitamin K1 (ng/mL) | 0.14 (0.36) | 0.92 (1.31) | -0.04 (0.26) | 0.011 |
| 8OHdG (ng/mL) | 44.05 (25.2) | -9.71 (16.94) | 1.16 (8.45) | 0.077 |
| Fecal 8OHdG (µg/mL) | 41.08 (100.6) | -23.45 (66.02) | -18.33 (54.27) | <0.001 |
| TNFα (pg/mL) | 163.67 (59.99) | -18.58 (59.61) | -8.94 (22.89) | 0.142 |
| IL6 (pg/mL) | 5.79 (3.17) | 1.88 (4.69) | 0.81 (6.3) | 0.011 |
| CRP (ng/mL) | 4661 (3479.98) | 0.7474 (3309.18) | -892.8 (3498.51) | 0.049 |
|  |  |  |  |  |
| Males (n=10) | -------- Mean (SD) -------- | | |  |
| Zonulin (ng/mL) | 4.07 (0.62) | 0.03 (0.65) | -0.26 (0.93) | <0.001 |
| Fecal Zonulin (ng/mL) | 5.87 (4.79) | -0.67 (5.66) | -0.81 (3.95) | 0.504 |
| LBP (ng/mL) | 4.63 (3.21) | -1.01 (3.69) | -0.36 (1.07) | 0.146 |
| ORM-1 (pg/mL) | 1221.26 (1197.96) | -409.22 (1149.86) | 78.26 (708.83) | 0.623 |
| Vitamin K1 (ng/mL) | 0.03 (0.1) | 0.38 (0.55) | 0.1 (0.36) | <0.001 |
| 8OHdG (ng/mL) | 35.29 (22.3) | -7.97 (18.08) | 0.51 (5.91) | 0.316 |
| Fecal 8OHdG (µg/mL) | 14.05 (29.14) | 1.45 (3.23) | -3.54 (9.04) | <0.001 |
| TNFα (pg/mL) | 145.16 (13.79) | -31.95 (36.14) | 7.6 (44.88) | 0.078 |
| IL6 (pg/mL) | 5.35 (3.81) | 0.94 (2.39) | 0.84 (7.8) | 0.001 |
| CRP (ng/mL) | 3261 (5906.45) | -1.9236 (6305.8) | -1078.4 (5759.44) | 0.693 |

Additional file 4: Table S1. Biomarkers of each sex (n=10 for both) participating in a high green leafy vegetable dietary intervention.

Bold values indicate significant differences between changes during intervention period vs. control period
