# Supplementary material for: Plasma and fecal zonulin are not altered by a high green leafy vegetable dietary intervention: secondary analysis of a randomized control crossover trial
Source: BMC Gastroenterol. 2022 Apr 12;22:184. doi: 10.1186/s12876-022-02248-3 (PMC9004007; doi:10.1186/s12876-022-02248-3)
Supplement: Supplementary file 5 — Additional file 5: Table S2. Select nutrient values of each sex (n=10 for both) participating in a high green leafy vegetable dietary intervention. [file 12876_2022_2248_MOESM5_ESM.docx]

| Females | -------- Mean (SD) -------- | | |  |
| --- | --- | --- | --- | --- |
| Energy (kcal) | 1870 (730.86) | -248.87 (745.64) | -39.49 (944.43) | 0.652 |
| Protein (g) | 71.61 (22.88) | -0.11 (18.13) | -9.41 (43.16) | 0.537 |
| Fat (g) | 76.43 (32.61) | -6.62 (39.58) | -3.27 (46.39) | 0.880 |
| Carb (g) | 227.26 (102.02) | -46.41 (112.61) | 15.03 (101.19) | 0.302 |
| Sugar (g) | 99.1 (55.44) | -26.08 (56.79) | 14.35 (56.52) | 0.197 |
| Fiber (g) | 16.33 (9.75) | 0.45 (7.35) | 2.88 (6.33) | 0.513 |
| Vitamin B12 (mcg) | 4.3 (2.72) | 1.27 (3.4) | -1.59 (2.02) | 0.022 |
| Vitamin K (mcg) | 125.69 (108.83) | 624.13 (766.22) | 37.2 (119.18) | 0.039 |
| α-linolenic acid (g) | 1.61 (1.18) | -0.69 (1.67) | 0.27 (0.85) | 0.167 |
| EPA (g) | 0.01 (0.01) | -0.01 (0.02) | 0 (0.02) | 0.399 |
| DHA (g) | 0.02 (0.02) | -0.02 (0.04) | 0 (0.03) | 0.189 |
| Dark GLV (cup) | 0.2 (0.28) | 0.77 (0.94) | 0.1 (0.42) | 0.065 |
| Whole grains (oz) | 0.74 (0.9) | -0.14 (0.91) | 0.37 (0.53) | 0.210 |
| Refined grains (oz) | 4.73 (2.55) | -0.21 (3.72) | -0.65 (3.76) | 0.786 |
| Meat total (oz) | 3.44 (2.23) | 0.11 (2.68) | -0.67 (3.5) | 0.601 |
| Red meat (oz) | 1.63 (1.67) | 0.08 (1.6) | -0.81 (1.47) | 0.326 |
| Cured meat (oz) | 0.52 (0.73) | 0.27 (0.69) | 0.16 (1.63) | 0.868 |
|  |  |  |  |  |
| Males | -------- Mean (SD) -------- | | |  |
| Energy (kcal) | 2262.15 (344.64) | 181.63 (1179.96) | 253.38 (1277.79) | 0.905 |
| Protein (g) | 95.39 (23.74) | 9.47 (52.79) | 10.45 (46.75) | 0.964 |
| Fat (g) | 98.41 (19.07) | 10.14 (72.43) | 5.48 (50.57) | 0.877 |
| Carb (g) | 252.55 (48.05) | 10.15 (105.28) | 32.34 (174.91) | 0.750 |
| Sugar (g) | 107.66 (44) | 24.75 (44.57) | 35.93 (118.05) | 0.772 |
| Fiber (g) | 16.83 (6.33) | 1.45 (10.53) | -2.29 (7.7) | 0.197 |
| Vitamin B12 (mcg) | 9.78 (8.9) | 0.35 (3.44) | 3.85 (11.08) | 0.297 |
| Vitamin K (mcg) | 140.29 (133.56) | 568.31 (353.36) | -82.32 (119.86) | <0.001 |
| α-linolenic acid (g) | 2.48 (1.44) | 0.54 (1.54) | -0.3 (1.15) | 0.101 |
| EPA (g) | 0.02 (0.02) | 0.01 (0.07) | 0.01 (0.03) | 0.912 |
| DHA (g) | 0.03 (0.04) | 0.01 (0.13) | 0.03 (0.09) | 0.733 |
| Dark GLV (cup) | 1.84 (0.98) | 0.41 (1) | -0.53 (0.83) | 0.018 |
| Whole grains (oz) | 0.12 (0.21) | 0.71 (0.28) | -0.14 (0.18) | <0.001 |
| Refined grains (oz) | 1.07 (0.97) | -0.58 (0.79) | -0.42 (0.69) | 0.576 |
| Meat total (oz) | 5.6 (1.21) | 0.23 (4.43) | 0.18 (4.25) | 0.983 |
| Red meat (oz) | 6.11 (3.05) | 0.92 (4.31) | 0.65 (3.44) | 0.899 |
| Cured meat (oz) | 1.97 (2.26) | 2 (2.73) | 0.22 (1.58) | 0.155 |
| Energy (kcal) | 1.54 (1.45) | -0.93 (1.71) | -0.47 (2.54) | 0.591 |

Additional file 5: Table S2. Select nutrient values of each sex (n=10 for both) participating in a high green leafy vegetable dietary intervention.

Bold values indicate significant differences between changes during intervention period vs. control period
